# Precision Dosing of Doxapram in Preterm Infants Using Continuous Pharmacodynamic Data and Model-Based Pharmacokinetics: An Illustrative Case Series

**DOI:** 10.3389/fphar.2020.00665

**Published:** 2020-05-12

**Authors:** Jarinda A. Poppe, Willem van Weteringen, Lotte L. G. Sebek, Catherijne A. J. Knibbe, Irwin K. M. Reiss, Sinno H. P. Simons, Robert B. Flint

**Affiliations:** ^1^ Department of Pediatrics, Division of Neonatology, Erasmus University Medical Center—Sophia Children's Hospital, University Medical Center Rotterdam, Rotterdam, Netherlands; ^2^ Department of Pediatric Surgery, Erasmus University Medical Center—Sophia Children's Hospital, University Medical Center Rotterdam, Rotterdam, Netherlands; ^3^ Department of Hospital Pharmacy, Erasmus University Medical Center, University Medical Center Rotterdam, Rotterdam, Netherlands; ^4^ Systems Biomedicine and Pharmacology, Leiden Academic Center for Drug Research, Leiden University, Leiden, Netherlands; ^5^ Department of Clinical Pharmacy, St. Antonius Hospital, Nieuwegein, Netherlands

**Keywords:** precision dosing, doxapram, pharmacokinetic modelling, pharmacodynamics, preterm infants

## Abstract

**Introduction:**

Current drug dosing in preterm infants is standardized, mostly based on bodyweight. Still, covariates such as gestational and postnatal age may importantly alter pharmacokinetics and pharmacodynamics. Evaluation of drug therapy in these patients is very difficult because objective pharmacodynamic parameters are generally lacking. By integrating continuous physiological data with model-based drug exposure and data on adverse drug reactions (ADRs), we aimed to show the potential benefit for optimized individual pharmacotherapy.

**Materials and Methods:**

Continuous data on oxygen saturation (SpO_2_), fraction of inspired oxygen (FiO_2_) and composite parameters, including the SpO_2_/FiO_2_ ratio and the cumulative oxygen shortage under the 89% SpO_2_ limit, served as indicators for doxapram effectiveness. We analyzed these continuous effect data, integrated with doxapram exposure and ADR parameters, obtained in preterm infants around the start of doxapram therapy. The exposures to doxapram and the active metabolite keto-doxapram were simulated using a population pharmacokinetic model. Infants were selected and retrospectively compared on the indication to start doxapram, the first response to doxapram, a potential dose-response relationship, and the administered dosage over time. Recommendations were made for individual improvements of therapy.

**Results:**

We provide eight cases of continuous doxapram administration that illustrate a correct and incorrect indication to start doxapram, responders and non-responders to therapy, and unnecessary over-exposure with ADRs. Recommendations for improvement of therapy include: objective evaluation of added effect of doxapram after start, prevention of overdosing by earlier down-titration or termination of therapy, and the prevention of hypoxia and agitation by measuring specific parameters at strategical time-points.

**Conclusion:**

Real-time and non-invasive effect monitoring of drug therapy combined with model-based exposure provides relevant information to clinicians and can importantly improve therapy. The variability between and within patients emphasizes the importance of individual, objective evaluation of pharmacotherapy. These measurements, together with data on ADRs, allow for precision medicine in neonatology that should be brought to the bedside.

## Introduction

Most drugs are off-label for use in preterm infants and administered according to standardized weight-based dosing regimens, as little is known about the pharmacokinetics (PK) and pharmacodynamics (PD) (Flint et al., 2018). Maturation of physiological processes that cause large inter- and intra-individual variability of PK and PD has hardly been taken into account (Allegaert and van den Anker, 2014). Parameters to measure PD in preterm infants are often poorly defined and depend on clinical observations, which results in subjective assessment of efficacy and safety. Altogether, these limitations lead to suboptimal pharmacotherapy in current neonatal care.

Neurological and respiratory underdevelopment in preterm infants often lead to temporary cessations of breathing, known as apnea of prematurity. The standard treatment of apnea of prematurity consists of caffeine (Schmidt et al., 2007) and non-invasive ventilatory support. If this treatment fails, doxapram can be added as off-label respiratory stimulant to avoid hypoxic periods which can lead to abnormal brain development (Poets et al., 2015). Invasive ventilation and the associated risk for bronchopulmonary dysplasia may be prevented (Vliegenthart et al., 2017b).

The effectiveness of doxapram is currently assessed by intermittent interpretation of vital sign alarms and nursing reports. This type of alarm interpretation by clinicians has been found inconsistent when compared to objectively analyzed monitor data (Brockmann et al., 2013). Continuous data on arterial oxygen saturation (SpO_2_) and administered fraction of inspired oxygen (FiO_2_) recently showed potential in assessing the PD of doxapram (Flint et al., 2017b; Poppe et al., 2019). Especially composite parameters that reflect oxygen need (SpO_2_/FiO_2_ ratio) and hypoxia (oxygen shortage under the 89% limit) were found strong indicators of therapy effect (Poppe et al., 2019).

Information on individual doxapram exposure during treatment could further improve pharmacotherapy. In the absence of evidence to support a target plasma concentration, the burden of invasive blood collections for measurement of doxapram plasma concentrations is not rational. To overcome this limitation, a population PK model could serve to simulate the individual exposure to doxapram and its active metabolite keto-doxapram (Flint et al., 2019). This simulated exposure could inform on the variation of clearance with gestational and postnatal age, and its influence on time to reach a steady state concentration following therapy start and dose adjustments.

Adverse drug reactions (ADRs) also need to be taken into account to titrate doxapram within the individual patient's therapeutic window. Several ADRs of doxapram have been reported in preterm infants, including QT interval lengthening (Maillard et al., 2001) possibly resulting in an atrioventricular heart block (De Villiers et al., 1998), gastrointestinal problems (Tay-Uyboco et al., 1991; Maillard et al., 2001), tachycardia (Barbe et al., 1999), increased electro-encephalographic activity and less sleep-wake cycling (Czaba-Hnizdo et al., 2014), irritability and agitation (Tay-Uyboco et al., 1991; Barbe et al., 1999), and hypokalemia (Fischer et al., 2013; Shimokaze et al., 2018).

Integrating the continuous effect data with model-based exposure to doxapram and keto-doxapram, ADR data and patient characteristics provides the opportunity for model-informed individual drug dosing. The aim of this study was to provide these integrated data for individual patients and to show the potential improvements for doxapram therapy.

## Materials and Methods

### Patient Population and Data Collection

A subset of patients from a cohort of 61 preterm infants, described in an earlier study (Poppe et al., 2019), was selected for this study. These patients provide various illustrative patient profiles with respect to the indication, the first response to doxapram start, the dosing regimen and the dose-response relationship of doxapram therapy. Patient characteristics were collected, including the gestational age, postnatal age at therapy start and post menstrual age at therapy start. Information on the FiO_2_, respiratory support mode, doxapram dosages, route of administration, and doxapram infusion rates was retrieved from the electronic patient data management system (Picis Clinical Solutions, Inc., Wakefield, MA, USA). The SpO_2_, reflecting effectiveness, was collected (1 Hz) from bedside monitors (Dräger, Lübeck, Germany). Collected data on ADRs included heart rate (HR), COMFORTneo scale, Numeric Rating Scale for agitation (NRS agitation), and potassium serum levels. Information on HR was collected (1 Hz) from the bedside monitors. Information on the COMFORTneo scale, NRS agitation, and potassium serum levels was extracted from the electronic medical records. Data were collected from 4 days before therapy start until 7 days after therapy start. The institutional ethics review board granted a waiver from approval according to the Medical Research Involving Human Subjects Act (WMO) in the Netherlands (MEC-2018-1106).

### Adverse Drug Reactions

Available data on ADRs of doxapram included COMFORTneo scale and NRS agitation as a measure of agitation, and heart rate to determine tachycardia. Events of agitation were defined as an NRS agitation ≥4, and as a COMFORTneo scale ≥14 (van Dijk et al., 2009) in combination with NRS agitation ≥4. The HR was compared visually with the HR trend in the 4 days before therapy start.

### Doxapram Treatment and Model-Based PK Simulations

The selected patients were treated according to the local clinical treatment policy, which states that the attending physicians can decide to start doxapram treatment at their own discretion. At therapy start a loading dose of 2.5 mg/kg in 15 min could be given, followed by a proposed maintenance dose of 2.0 mg/kg/h by intravenous infusion or continuous gastro-intestinal administration of the intravenous solution *via* a nasogastric tube if enteral feeding was tolerated. The dosage was increased or decreased by 0.5 mg/kg/h when indicated by the physician. For each individual patient, the concentration-time profile was simulated using a population PK model for doxapram and keto-doxapram in preterm infants with NONMEM V.7.3 (ICON Development Solutions, Ellicott City, MD, USA) (Flint et al., 2019). The final pharmacokinetic parameter estimates are given in **Supplementary Table 1**. The PK of doxapram was best described by a two-compartment model with intra-individual variability on clearance of doxapram through other routes than formation into keto-doxapram and central volume of distribution. Keto-doxapram concentrations were also best described by a two-compartment model. Postnatal age and gestational age were found to be the best predictors of maturation of clearance, describing both the elimination of doxapram through other routes than formation to keto-doxapram (CLD) and the formation clearance of keto-doxapram from doxapram (CLD-KD). For an individual of 0.95 kg, gestational age of 25.9 weeks and postnatal age of 17 days, CLD-KD was 0.096 L/h [relative standard error (RSE) 22%] and CLD was 0.493 L/h (RSE 13%). Oral bioavailability was estimated at 74%. The exposures to doxapram and keto-doxapram were simulated in 1-h timeframes.

### Data Analysis

The monitor data, the SpO_2_ and HR, were processed in median per hour using R software (version 3.5.3., Inc., Boston, MA, USA). The depth x time <89% SpO_2_ (cumulative oxygen shortage <89% limit that reflects hypoxia) was calculated and processed as absolute number per hour. From these absolute numbers the mean oxygen shortage per second (%/s) was calculated in a particular hour. The SpO_2_/FiO_2_ ratio was calculated per second to reflect the oxygen need. This ratio represents the SpO_2_ corrected for the FiO_2_, which is the main respiratory support aside pharmacotherapy. The ratio was processed as median per hour in the analysis.

### Evaluation Strategy

The selected patients were evaluated visually on the indication, the first response to doxapram, the dosing regimen and the occurrence of ADRs. We re-evaluated the clinical decisions in retrospect at certain time points (T) and suggested possible improvements of therapy if these data would have been available at the bedside for the attending clinicians. The indication was assessed based on deterioration of the respiratory condition before therapy start, the level of hypoxia, and the possibilities to improve the respiratory condition. The first response was evaluated by the change in hypoxia, oxygen supply (reflected by the FiO_2_) and the oxygen need directly after doxapram start. A patient was classified as responder if hypoxia, oxygen supply or oxygen need had decreased. The dose-response relationship was evaluated as a change in the respiratory condition after dose adjustments. If no dose-response relationship was found or minimal dose adjustments had been made, the dosing regimen was defined as overtreatment. Therapy was defined as failure, if mechanical ventilation was required after therapy stop.

## Results

### Patient Characteristics

Eight patients were selected out of a cohort of 61 preterm infants who received doxapram therapy. The gestational age of these selected patients varied between 25.1 and 28.0 weeks, the postmenstrual age at therapy start varied between 27.6 and 32.8 weeks, and the postnatal age at therapy start varied between 8.5 and 34.0 days (**Table 1**). In five patients, the mechanical ventilation had been stopped within 24 h before doxapram start. Therapy was successful in five of the eight patients.

**Table 1:** Baseline characteristics of the subset (n=8).

Patient	GA (weeks)	PMA (weeks)	PNA (days)	MV	Therapy outcome^a^	ROA	Loading dose	Maintenance dose (mg/kg/h)^b^
A	25.1	27.6	17.1	No	Success	Oral	Yes	1.3 ± 0.4
B	26.1	27.8	11.4	Yes	Failure	IV	Yes	1.8 ± 0.4
C	27.0	30.1	21.7	No	Success	IV	Yes	1.9 ± 0.1
D	26.9	31.7	34.0	No	Success	Oral	No	1.3 ± 0.5
E	25.6	27.6	14.1	Yes	Failure	Oral	No	1.4 ± 0.5
F	25.3	27.7	17.0	Yes	Failure	IV	Yes	2.0 ± 0.0
G	28.0	32.8	33.6	Yes	Success	IV	No	1.1 ± 0.3
H	27.6	28.8	8.5	Yes	Success	Oral	Yes	0.9 ± 0.4

GA, Gestational age; PMA, Postmenstrual age at start of doxapram therapy; PNA, Postnatal age at start of doxapram therapy; MV, Mechanical ventilation in the 24 h before doxapram start; ROA, Route of administration at start of doxapram therapy; IV, Intravenous.

a Therapy failure was defined as the need for mechanical ventilation after therapy stop.

b Maintenance dose during the study period (mean ± SD).

### Integration of Effect Data, Model-Based Exposure and Adverse Drug Reactions

**Figure 1:**
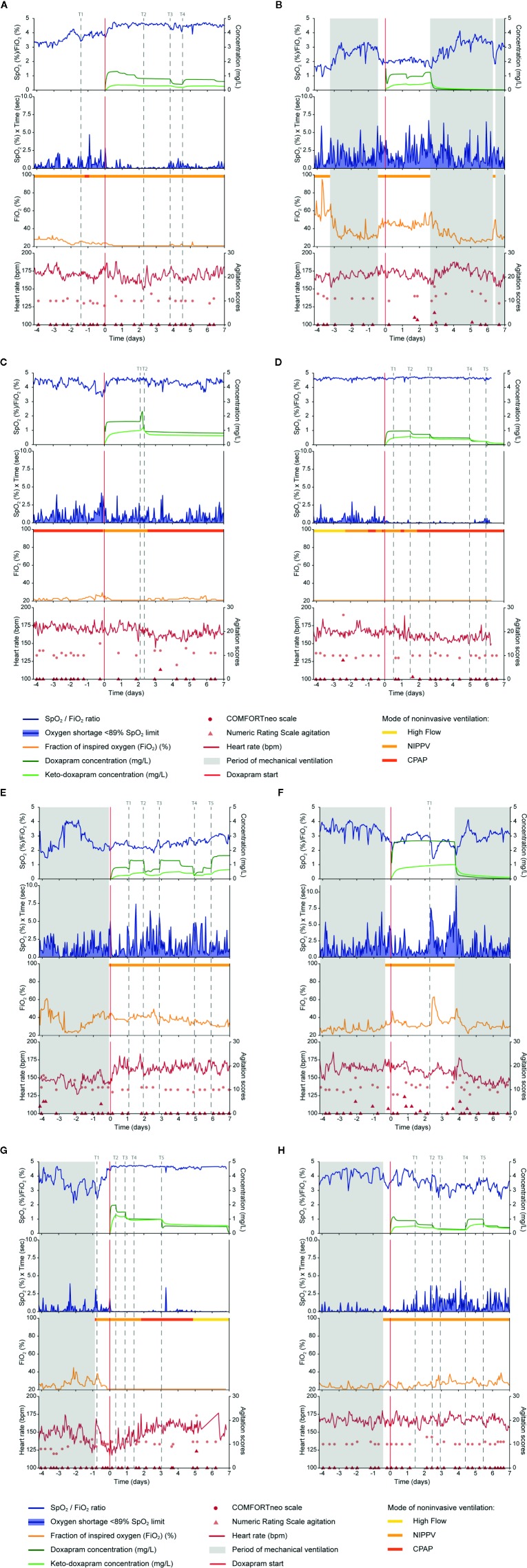
Data on doxapram effect, exposure and ADRs are visualized of the eight selected preterm infants **(A–H)**. Doxapram effect is reflected by the oxygen saturation (SpO_2_), fraction of inspired oxygen (FiO_2_), the oxygen shortage <89% SpO_2_ limit, and the SpO_2_/FiO_2_ ratio. Doxapram and keto-doxapram exposures were simulated from a population pharmacokinetic model. The NRS agitation, COMFORTneo scale, and heart rate reflected the adverse drug reactions agitation and tachycardia. Specific time-points were marked (T). ADRs, adverse drug reactions; NRS agitation, Numeric Rating Scale agitation.

For all eight cases (A–H), we visualized the effect data, exposure and ADR parameters before and after doxapram start (**Figure 1**). Doxapram and keto-doxapram exposures were provided by model-based simulated plasma concentrations after therapy start. The dose adaptations over time were reflected in the exposure. The suggested possible improvements of therapy are presented in **Table 2**.

**Table 2:** Overview of the retrospective evaluation and recommendations for improvements.

Patient	Indication	First response	Dosing regimen	ADRs^a^	Potential improvements with bedside availability
A	Correct	Responder	Dose-response relationship	Increased heart rate variability	Start therapy earlier to prevent hypoxia and decrease dosage every 12 h to prevent overtreatment
B	Correct	Non-responder	Overtreatment	Increased agitation	Stop therapy earlier to prevent hypoxia and overtreatment with ADRs
C	Correct	Responder	Dose-response relationship	No ADRs observed	Evaluate therapy effect at least every 12 h and increase dosage to prevent hypoxia
D	Incorrect	Responder	Overtreatment	No ADRs observed	Do not start doxapram therapy, and decrease dosage every 12 h to prevent overtreatment
E	Correct	Potential responder	Potential dose-response relationship	Increased heart rate	Stop therapy earlier to prevent hypoxia and overtreatment with ADRs
F	Correct	Non-responder	Overtreatment	Increased agitation	Stop therapy earlier to prevent hypoxia and overtreatment with ADRs
G	Probably correct	Responder	Overtreatment	No ADRs observed	Decrease dosage every 12 h to prevent overtreatment
H	Probably incorrect	Potential responder	Potential dose-response relationship	No ADRs observed	Evaluate therapy effect at least every 12 h to prevent hypoxia

a Possible adverse drug reaction (ADR).

#### The Indication of Doxapram Therapy

In the hour before therapy start, the cumulative oxygen shortage <89% limit varied between 0.3 and 5.1%/s; the FiO_2_ varied between 21.0 and 47.9%; and the SpO_2_/FiO_2_ ratio varied between 1.9 and 4.6 (**Table 3**).

**Table 3:** The individual values at 1 h before doxapram, and the changes from 1 h before until 4 h after doxapram start.

Patient	Oxygen shortage (%/s)^a^	FiO_2_ (%)^a^	SpO_2_/FiO_2_ ratio^a^	∆ Oxygen shortage (%/s)^b^	∆ FiO_2_ (%)^b^	∆ SpO_2_/FiO_2_ ratio^b^
A	2.9	24.0	3.67	-2.8	-1.0	0.58
B	2.1	47.9	1.90	-1.7	1.9	0.04
C	2.2	26.0	3.58	-2.1	-2.0	0.54
D	0.3	21.0	4.61	-0.2	0.0	-0.03
E	5.1	41.5	2.08	-5.1	0.3	0.19
F	4.3	34.3	2.56	-4.1	4.3	-0.14
G	1.9	21.0	4.38	-1.8	0.0	0.27
H	0.3	26.0	3.57	-0.0	-1.5	0.14

a One hour before doxapram start.

b Change from 1 h before until 4 h after doxapram start.

In five patients the level of hypoxia had increased before therapy start, with an increased or equal oxygen supply and oxygen need (Patients A, B, C, E, F), which indicates that the patients' conditions deteriorated and the indication to start therapy could be justified in retrospect. The extubation within the 24 h before therapy start in three of these patients (Patients B, E, F) had resulted in an increased level of hypoxia, oxygen supply, and oxygen need. This respiratory deterioration was also observed in Patient G after extubation, although the level of hypoxia and oxygen supply decreased again before therapy start at T1. Doxapram was started despite these respiratory improvements, and the indication to start doxapram could be questioned. Two other patients (Patients D, H) had no increased level of hypoxia before doxapram start. In Patient D, the oxygen supply and oxygen need had not increased before therapy start, and the level of hypoxia was minimal. In Patient H, oxygen supply, and consequently oxygen need, had increased before therapy start. As doxapram was started to prevent hypoxia, the indication for doxapram seemed incorrect of these two patients (Patients D, H).

#### Detection of Responders and Non-Responders

The change in the cumulative oxygen shortage <89% limit between the time-points 1 h before and 4 h after doxapram start varied between -5.1 and -0.0%/s. The change in the FiO_2_ varied between -2.0 and 4.2%; the change in the SpO_2_/FiO_2_ ratio varied between -0.1 and 0.6. The individual changes are presented in **Table 3**.

We classified four patients as responder to doxapram therapy, based on the decreased level of hypoxia after therapy start (Patients A, C, D, G). In two of these (Patients A, C), the respiratory improvement was also reflected in a decreased oxygen supply and oxygen need. In Patient D, the effects on oxygen supply and oxygen need could not be determined as both were already minimal before therapy start. A small effect of doxapram was observed in Patient G, although improvement of the respiratory condition already started already before therapy start. We classified two patients as potential responder (Patients E, H), based on a decreased oxygen supply and oxygen need. The level of hypoxia decreased slightly in Patient E, while the level of hypoxia was already low before therapy start in Patient H. Classification of this patient was also impeded due to the large variability in the oxygen supply and oxygen need. We classified the two remaining patients as non-responder (Patients B, F). The decrease in the level of hypoxia was questionable in both patients, and the oxygen supply and oxygen need increased (Patient F) or remained equal (Patient B) directly after therapy start.

#### Dose-Response Relationship

A dose-response relationship was found in two patients (Patients A, C). A decreased exposure at time-point T3 in Patient A and at T2 in Patient C resulted in a deteriorated respiratory condition with an increased hypoxia and a larger variability in the oxygen need. The increased exposure at T4 in Patient A and at T1 in Patient C decreased the level of hypoxia and the variability in the oxygen need. Despite multiple dose adjustments in two other patients (Patients E, H), a dose-response relationship in these cases was questionable. The increased exposure at T1 in Patient E did not decrease the hypoxia, although the hypoxia decreased slightly after the increased exposure at T3 and T5, and increased after a decreasing exposure at T2 and T4. The hypoxia in Patient H increased after a decreasing exposure at T1, T2, and T5, and decreased after an increasing exposure at T4. The large variability in the oxygen supply and in the oxygen need complicates the evaluation of the dose-response relationship in Patient H.

A dose-response relationship was not found in the four remaining patients (Patients B, D, F, G). In two of these patients (Patients B, F), minimal dose adjustments had been made during the treatment period, which made the evaluation of a dose-response relationship impossible. Regarding the two other patients, we found no effect of the exposure decrease after down-titration at T2, T3, T4, and T5 in Patient D and at T2, T3, and T5 in Patient G. Those four patients have probably been overtreated with doxapram (Patients B, D, F, G).

#### Adverse Drug Reactions

A remarkable increase in the HR was observed in Patient E after doxapram start. The HR increased directly after doxapram start and remained above the baseline HR before therapy start. The other patients showed no increase in the HR, although the HR variability increased in Patient A after doxapram start. Two patients showed some agitation during doxapram therapy (Patients B, F). The other four patients showed no ADRs (Patients C, D, G, H), although in Patient C the HR had decreased after a decreased exposure.

### Potential Doxapram Therapy Improvements per Patient

#### Patient A

Doxapram therapy could already have been started at T1 to avoid the hypoxia between T1 and actual therapy start. After day 2 of doxapram, the dosage was not further down-titrated for another 2 days. The dosage should have been decreased around T2 to prevent overdosing. Therapy should have been evaluated more strictly, every 12 h, and the dosage should have been decreased sooner (**Figure 1A**).

#### Patient B

Judging from the effect data, mechanical ventilation should have started a day after therapy start, as no response to doxapram was seen. This could have prevented doxapram overtreatment, the agitation around day 2 and unnecessary hypoxia (**Figure 1B**).

#### Patient C

Although the oxygen supply and the variability of the oxygen need increased after a dose reduction at T2, no dose adjustments were made for at least 5 days. Therapy should have been evaluated at least every 12 h, and the dosage should have been increased to prevent hypoxia (**Figure 1C**).

#### Patient D

The continuous effect data showed no reason to start doxapram therapy, and bedside availability of the data could have prevented this. The first down-titration was done at T2, 1.5 days after therapy start. The doxapram dosage should have been down-titrated every 12 h, starting at T1. This would likely have prevented unnecessary long doxapram therapy (**Figure 1D**).

#### Patient E

The HR increased directly after therapy start in this patient. As periods of hypoxia still occurred during doxapram therapy and the HR increased, mechanical ventilation should have been started earlier to prevent hypoxia and ADRs (**Figure 1E**).

#### Patient F

The level of agitation was scored higher during doxapram therapy and the exposure to doxapram was relatively high. Judging from these data, we suggest that mechanical ventilation should have been resumed earlier, at least before T1, to prevent hypoxia and ADRs (**Figure 1F**).

#### Patient G

The dosage should have been decreased again at T4 to prevent overtreatment. In general, we would suggest to decrease the dosage every 12 h if a patient's respiratory condition has not deteriorated (**Figure 1G**).

#### Patient H

Due to the large variability in the effect parameters the indication, first response and dose-response relationship were difficult to evaluate. The dosage should have been increased again at T3 and the therapy effect should have been evaluated more strictly, at least every 12 h, in the following treatment period (**Figure 1H**).

## Discussion

This study showed that pharmacotherapy may be improved with the real-time availability of continuous monitor data integrated with model-informed exposure data, and ADR parameters. Using this approach, we could suggest several improvements for doxapram therapy for eight illustrative preterm infants. Access to these data at the bedside can support clinicians to adequately indicate and evaluate therapy. This may reduce overdosing, unnecessary long treatment, suboptimal treatment and the occurrence of ADRs. The inter-individual differences in the observed data trends and patterns advocate an individualized approach. Real-time effect evaluation supports the attending physician in identifying patients for whom doxapram will be effective.

In all studies reporting apnea rate in preterm infants, doxapram therapy led to fewer cases of apnea (Vliegenthart et al., 2017a). A positive effect of doxapram and—to a lesser extent of keto-doxapram—on the respiratory drive was also found in newborn lambs (Bairam et al., 1990). Two retrospective studies concluded that doxapram therapy may avoid mechanical ventilation (Prins et al., 2013; Flint et al., 2017a). In our earlier studies, doxapram therapy had a positive effect on the SpO_2_, the applied FiO_2_ (Flint et al., 2017b), the oxygen need (SpO_2_/FiO_2_ ratio), and hypoxia (cumulative oxygen shortage under the 89% limit) (Poppe et al., 2019). The present study is the first in which doxapram therapy effects were continuously evaluated in individual patients.

If integrated data would have been continuously available, several patients could have been prevented from unnecessarily long doxapram treatment (Patients B, D, F, G), hypoxia (Patients B, C, E, F, H), and overdosing (Patient F). Even without a doxapram target concentration, visualizing the exposures to doxapram and keto-doxapram will be relevant to monitor dosage adjustments and an expected new steady state. The exposure could also be predicted a few hours ahead to support dosage adjustments. In this study, the simulated data from the PK model could only be used to explain, and not to predict the clinical outcomes, as data were collected retrospectively. Furthermore, visualized exposure permits detection of the individual relationships with therapy effectiveness or ADRs, which could serve as an individual target for therapy. An individual target is in particular important for doxapram therapy as dosing regimens have been based on body weight, while exposure is related to the gestational age and postnatal age (Flint et al., 2019). A measured plasma concentration of doxapram and keto-doxapram would make the simulated exposure from the PK model more accurate as inter-patient variability is taken into account.

In addition to the effectiveness and drug exposure, our study suggests the integration of ADRs into routine evaluation of pharmacotherapy. Although some ADRs of doxapram have been described in neonates (Tay-Uyboco et al., 1991; De Villiers et al., 1998; Barbe et al., 1999; Maillard et al., 2001; Fischer et al., 2013; Czaba-Hnizdo et al., 2014; Shimokaze et al., 2018), the results of these studies were not conclusive, and sample sizes were small in most studies. In our study, we found that ADR parameters were underreported, in part because they had not been specifically registered to assess ADRs of doxapram. The occurrence of hypokalemia could not be evaluated, for example, because potassium serum levels in the study period were not available for all eight patients. This can be explained by unawareness of clinicians about drug specific ADRs. Next to under detection, underreporting of ADRs is also a common problem, as well in adult clinical care, and could be improved with automated ADR detection systems (McMaster et al., 2019).

Causality between pharmacotherapy and a potential ADR is often difficult in preterm infants as most potential ADRs are difficult to distinguish from common complications related to preterm birth itself (Allegaert and van den Anker, 2015). Drug specific and routine assessment of ADRs has not been implemented in neonatal intensive care treatment, although important steps have recently been made to interpret possible ADRs (Salaets et al., 2019). Defining adequate ADR parameters and registering these around each dose adjustment would improve ADR evaluation. The projection of possible ADRs likely improves the recognition and may create more awareness to evaluate and register ADRs.

The large differences in response to doxapram between the eight selected patients illustrate the need to explore individual risk-benefit profiles. Some patients showed respiratory improvement directly after doxapram start, while others did not show a response at all. Identifying predictors of non-response to doxapram therapy can prevent unnecessary doxapram treatment and therewith suboptimal therapy and possible harm. The first response to doxapram therapy can already inform the clinician on therapy outcome. Previously we have found that the SpO_2_/FiO_2_ ratio in the 48 h around start of doxapram, corrected for postnatal age and mechanical ventilation before therapy start, was a predictor for therapy failure or success (Poppe et al., 2019). Clearance, and the related exposure and effect, may differ from the general preterm population in, for instance, those small for gestational age with different severity of illness and co-medication (Allegaert et al., 2017).

Doxapram is indicated as an additional therapy to non-invasive ventilation, oxygen supply and caffeine treatment. In part, all therapies share the same effect and aim to protect the newborn against hypoxia. These co-interventions need, therefore, to be taken into account in the effect evaluation of doxapram therapy. The FiO_2_ was already used to assess the doxapram effects, and the effect of non-invasive mode seems limited (**Figure 1**). In Patient C (**Figure 1C**), however, it is challenging to distinguish the effect of doxapram from the respiratory support as CPAP was switched to NIPPV just before doxapram start. Data on apneas could partly correct for the influence of these confounders, but the detection of apneas remains challenging and the definition varies between centers. Additionally, in the majority of patients no relationship could be observed between PK and PD. This finding points out that there are still unknown factors that influence the PK, PD, or both. The factors that change the respiratory status could be respiratory support and coexisting medical conditions such as infections, patent ductus arteriosus, and feeding problems. Identification of these factors is essential before individual dose titration for targeting a PD effect, based on a PKPD relationship, can be implemented into clinical practice.

Tachycardia is a potential ADR of both doxapram and caffeine. A previous study found more tachycardia in preterm infants with high caffeine dosage than in those with low caffeine dosage (Chen et al., 2018). All eight selected patients in the present study were treated with caffeine. To further improve the treatment of hypoxia, the administered caffeine dosages and the caffeine exposure should be integrated in the data as well. Also, pharmacokinetic interactions may occur with co-medication and influence the clearance of doxapram. Fluconazole, for example, which is used to protect preterm infants from fungal infections, is an inhibitor of CYP3A4 and will decrease the clearance of doxapram that is mainly metabolized by this enzyme (Ogawa et al., 2015).

Real-time monitoring with integrated data may also be used for other drugs. Vinks et al. recently provided a real-time bedside decision support system with drug exposure and response for morphine administration in neonatal pain management (Vinks et al., 2019). The safety of morphine could be monitored using continuous physiological data (Hartley et al., 2018). For all drugs and indications objective PD parameters are needed to apply this concept. This is challenging in drugs such as fentanyl and analgesia, but new developments in continuous pain measurements may be helpful (Moultrie et al., 2017).

The continuous collection of data on effect, exposure and ADRs could be suitable for automatic dosing systems. Our findings urge caution, however. We found large differences in the PK and PD of doxapram within and between patients, which preclude defining the optimal effect and target concentration. Both the optimal effect and the target concentration are likely to change over time. Consequently, the dose-response relationship may change over time. Thus, the concept of automated adjustments on the basis of effect parameters still belongs to a distant future. Until then, the responsibility for both interpretation of all data streams and the adjustment of pharmacotherapy remains with the clinician.

**Figure 2:**
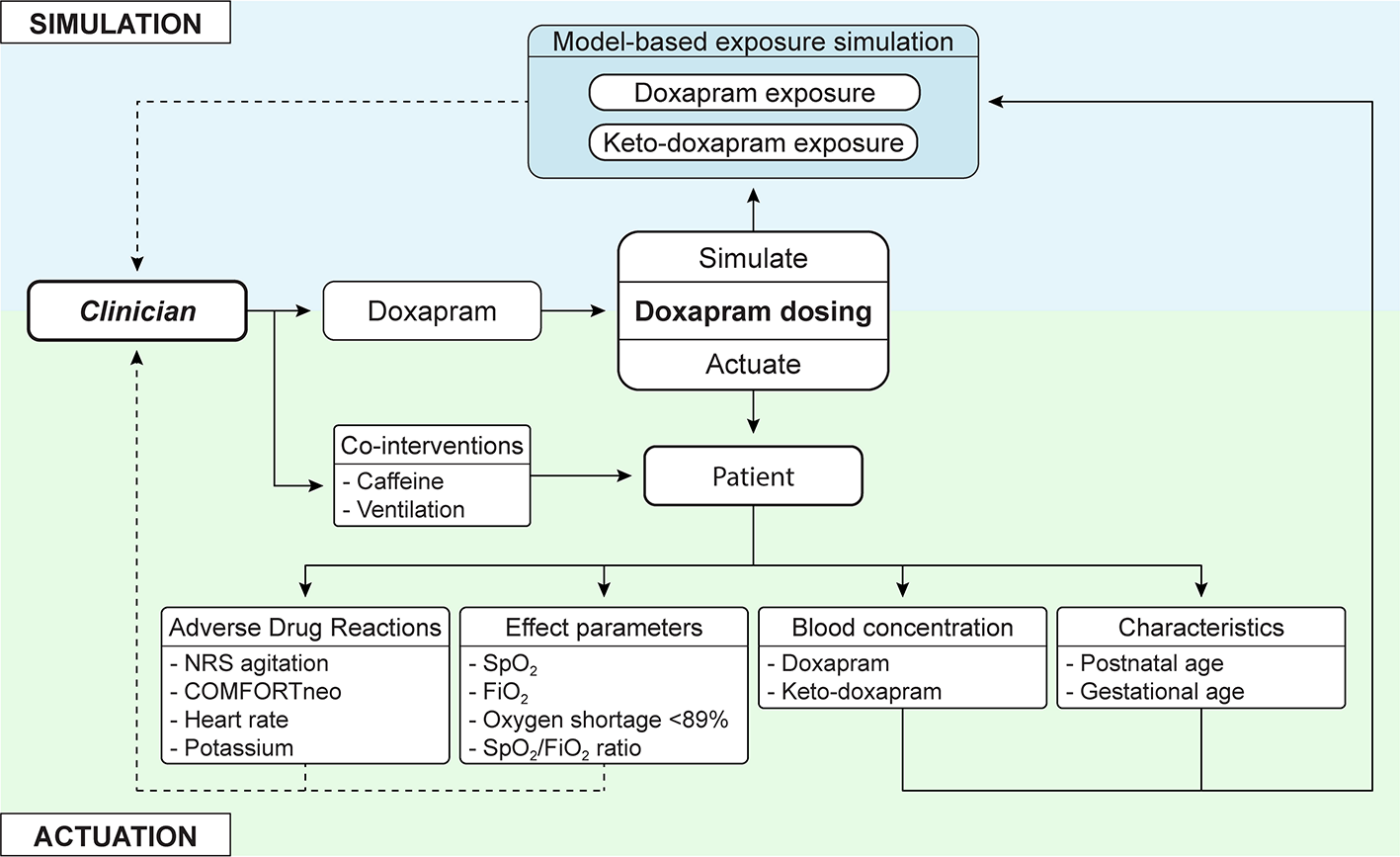
In addition to current clinical care, in which the clinician actuates a dosing regimen, model-based simulated exposure of considered dose adjustments is added. This offers simulation of doxapram and keto-doxapram exposures, adjusted to the individual patient. The interpretation of this information and actuation of changes still resides with the clinician, who can be presented extensive information on adverse drug reactions and effect parameters. NRS agitation, Numeric Rating Scale agitation; SpO_2_, oxygen saturation; FiO_2_, fraction of inspired oxygen.

In clinical practice, the implementation of individualized evaluation of continuous data may lead to the following events. Imagine an extremely preterm infant who was extubated earlier for 12 h after several days of invasive ventilation. The infant shows frequent apneas, oxygen desaturations and is at risk for re-intubation. The clinician decides to start doxapram therapy, but the real-time effect data show no signs of improvement. The model-informed exposure indicates that it takes 8 h to reach steady state exposure. The monitor trend alerts, however, warn that the infant is more agitated with increased tachycardia even before the model-informed exposure has reached steady state. These data assist the clinician in making the decision to stop doxapram therapy and to re-intubate this individual infant. This case illustrates how simulation of the exposure resulting from a considered dose adjustment at the bedside is likely to be helpful in targeting blood concentrations in individual patients, avoiding ADRs and optimizing effectiveness, as illustrated in **Figure 2**.

## Conclusion

Current pharmacotherapy for preterm infants is suboptimal and could be improved by continuously collecting monitor data, model-informed exposure and ADR parameters. Data collection and evaluation of more doxapram patients in the future will improve the classification and recognition of responders and non-responders. The concept of individualized evaluation of continuous data should be implemented in clinical care.

## Data Availability Statement

The datasets generated for this study are available on request to the corresponding author.

## Ethics Statement

The studies involving human participants were reviewed and approved by Medisch Ethische Toetsings Commissie (METC) Erasmus MC. Written informed consent from the participants' legal guardian/next of kin was not required to participate in this study in accordance with the national legislation and the institutional requirements. Written informed consent was not obtained from the minor(s)' legal guardian/next of kin for the publication of any potentially identifiable images or data included in this article.

## Author Contributions

CK, IR, RF, SS, and JP contributed conception and design of the study. JP and RF organized the database. JP performed the statistical analysis. RF and JP wrote the first draft of the manuscript. SS, LS, RF, WW, and JP contributed to sections of the manuscript. All authors contributed to manuscript revision, read and approved the submitted version.

## Funding

This study was enabled by funding from the Sophia Foundation (Grant number: S18-27).

## Conflict of Interest

The authors declare that the research was conducted in the absence of any commercial or financial relationships that could be construed as a potential conflict of interest.

## References

[B1] Allegaert K. van den Anker J. N. (2014). Clinical pharmacology in neonates: small size, huge variability. Neonatology 105 (4), 344–349. 10.1159/000360648 24931327 PMC4111147

[B2] Allegaert K. van den Anker J. N. (2015). Adverse drug reactions in neonates and infants: a population-tailored approach is needed. Br. J. Clin. Pharmacol. 80 (4), 788–795. 10.1111/bcp.12430 24862557 PMC4594721

[B3] Allegaert K. Simons S. H. P. Tibboel D. Krekels E. H. Knibbe C. A. van den Anker J. N. (2017). Non-maturational covariates for dynamic systems pharmacology models in neonates, infants, and children: Filling the gaps beyond developmental pharmacology. Eur. J. Pharm. Sci. 109S, S27–S31. 10.1016/j.ejps.2017.05.023 28506866

[B4] Bairam A. Blanchard P. W. Mullahoo K. Beharry K. Laudignon N. Aranda J. V. (1990). Pharmacodynamic effects and pharmacokinetic profiles of keto-doxapram and doxapram in newborn lambs. Pediatr. Res. 28 (2), 142–146. 10.1203/00006450-199008000-00013 2395604

[B5] Barbe F. Hansen C. Badonnel Y. Legagneur H. Vert P. Boutroy M. J. (1999). Severe side effects and drug plasma concentrations in preterm infants treated with doxapram. Ther. Drug Monit. 21 (5), 547–552. 10.1097/00007691-199910000-00011 10519454

[B6] Brockmann P. E. Wiechers C. Pantalitschka T. Diebold J. Vagedes J. Poets C. F. (2013). Under-recognition of alarms in a neonatal intensive care unit. Arch. Dis. Child Fetal. Neonatal. Ed. 98 (6), F524–F527. 10.1136/archdischild-2012-303369 23716498

[B7] Chen J. Jin L. Chen X. (2018). Efficacy and Safety of Different Maintenance Doses of Caffeine Citrate for Treatment of Apnea in Premature Infants: A Systematic Review and Meta-Analysis. BioMed. Res. Int. 2018, 9061234. 10.1155/2018/9061234 30671477 PMC6323495

[B8] Czaba-Hnizdo C. Olischar M. Rona Z. Weninger M. Berger A. Klebermass-Schrehof K. (2014). Amplitude-integrated electroencephalography shows that doxapram influences the brain activity of preterm infants. Acta Paediatr. 103 (9), 922–927. 10.1111/apa.12681 24813556

[B9] De Villiers G. S. Walele A. Van der Merwe P. L. Kalis N. N. (1998). Second-degree atrioventricular heart block after doxapram administration. J. Pediatr. 133 (1), 149–150. 10.1016/S0022-3476(98)70197-0 9672531

[B10] Fischer C. Ferdynus C. Gouyon J. B. Semama D. S. (2013). Doxapram and hypokalaemia in very preterm infants. Arch. Dis. Child Fetal. Neonatal. Ed. 98 (5), F416–F418. 10.1136/archdischild-2012-303089 23448699

[B11] Flint R. Halbmeijer N. Meesters N. van Rosmalen J. Reiss I. van Dijk M. (2017a). Retrospective study shows that doxapram therapy avoided the need for endotracheal intubation in most premature neonates. Acta Paediatr. 106 (5), 733–739. 10.1111/apa.13761 28130789

[B12] Flint R. Weteringen W. V. Voller S. Poppe J. A. Koch B. C. Groot R. (2017b). Big data analyses for continuous evaluation of pharmacotherapy: A proof of principle with doxapram in preterm infants. Curr. Pharm. Des. 23 (38), 5919–5927. 10.2174/1381612823666170918121556 28925893

[B13] Flint R. B. van Beek F. Andriessen P. Zimmermann L. J. Liem K. D. Reiss I. K. M. (2018). Large differences in neonatal drug use between NICUs are common practice: time for consensus? Br. J. Clin. Pharmacol. 84 (6), 1313–1323. 10.1111/bcp.13563 29624207 PMC5980600

[B14] Flint R. B. Simons S. H. P. Andriessen P. Liem K. D. Degraeuwe P. L. J. Reiss I. K. M. (2019). O17 The bioavailability and maturing clearance of doxapram in preterm infants. Arch. Dis. Childhood 104 (6), e8–e8. 10.1136/archdischild-2019-esdppp.17 32698193

[B15] Hartley C. Moultrie F. Hoskin A. Green G. Monk V. Bell J. L. (2018). Analgesic efficacy and safety of morphine in the Procedural Pain in Premature Infants (Poppi) study: randomised placebo-controlled trial. Lancet 392 (10164), 2595–2605. 10.1016/S0140-6736(18)31813-0 30509743 PMC6294828

[B16] Maillard C. Boutroy M. J. Fresson J. Barbe F. Hascoet J. M. (2001). QT interval lengthening in premature infants treated with doxapram. Clin. Pharmacol. Ther. 70 (6), 540–545. 10.1016/S0009-9236(01)95877-1 11753270

[B17] McMaster C. Liew D. Keith C. Aminian P. Frauman A. (2019). A Machine-Learning Algorithm to Optimise Automated Adverse Drug Reaction Detection from Clinical Coding. Drug Saf. 42 (6), 721–725. 10.1007/s40264-018-00794-y 30725336

[B18] Moultrie F. Slater R. Hartley C. (2017). Improving the treatment of infant pain. Curr. Opin. Support Palliat. Care 11 (2), 112–117. 10.1097/SPC.0000000000000270 28375883 PMC5419813

[B19] Ogawa Y. Irikura M. Kobaru Y. Tomiyasu M. Kochiyama Y. Uriu M. (2015). Population pharmacokinetics of doxapram in low-birth-weight Japanese infants with apnea. Eur. J. Pediatr. 174 (4), 509–518. 10.1007/s00431-014-2416-1 25248340

[B20] Poets C. F. Roberts R. S. Schmidt B. Whyte R. K. Asztalos E. V. Bader D. (2015). Association Between Intermittent Hypoxemia or Bradycardia and Late Death or Disability in Extremely Preterm Infants. JAMA 314 (6), 595–603. 10.1001/jama.2015.8841 26262797

[B21] Poppe J. A. van Weteringen W. Völler S. Goos T. G. Reiss I. K. M. Simons S. H. P. (2019). P80 Objective pharmacodynamic evaluation of doxapram in preterm infants. Arch. Dis. Childhood 104 (6), e50–e50. 10.1136/archdischild-2019-esdppp.118

[B22] Prins S. A. Pans S. J. van Weissenbruch M. M. Walther F. J. Simons S. H. (2013). Doxapram use for apnoea of prematurity in neonatal intensive care. Int. J. Pediatr. 2013, 251047. 10.1155/2013/251047 24376463 PMC3860126

[B23] Salaets T. Turner M. A. Short M. Ward R. M. Hokuto I. Ariagno R. L. (2019). Development of a neonatal adverse event severity scale through a Delphi consensus approach. Arch. Dis. Child. 104 (12), 1167–1173. 10.1136/archdischild-2019-317399 31537552 PMC6943241

[B24] Schmidt B. Roberts R. S. Davis P. Doyle L. W. Barrington K. J. Ohlsson A. (2007). Long-term effects of caffeine therapy for apnea of prematurity. N. Engl. J. Med. 357 (19), 1893–1902. 10.1056/NEJMoa073679 17989382

[B25] Shimokaze T. Toyoshima K. Shibasaki J. Itani Y. (2018). Blood potassium and urine aldosterone after doxapram therapy for preterm infants. J. Perinatol. 38 (6), 702–707. 10.1038/s41372-018-0087-x 29515224

[B26] Tay-Uyboco J. Kwiatkowski K. Cates D. B. Seifert B. Hasan S. U. Rigatto H. (1991). Clinical and physiological responses to prolonged nasogastric administration of doxapram for apnea of prematurity. Biol. Neonate 59 (4), 190–200. 10.1159/000243342 2070020

[B27] van Dijk M. Roofthooft D. W. Anand K. J. Guldemond F. de Graaf J. Simons S. (2009). Taking up the challenge of measuring prolonged pain in (premature) neonates: the COMFORTneo scale seems promising. Clin. J. Pain 25 (7), 607–616. 10.1097/AJP.0b013e3181a5b52a 19692803

[B28] Vinks A. A. Punt N. C. Menke F. Kirkendall E. Butler D. Duggan T. J. (2019). Electronic Health Record-Embedded Decision Support Platform for Morphine Precision Dosing in Neonates. Clin. Pharmacol. Ther. 107 (1), 186–194. 10.1002/cpt.1684 31618453 PMC7378965

[B29] Vliegenthart R. J. Ten Hove C. H. Onland W. van Kaam A. H. (2017a). Doxapram Treatment for Apnea of Prematurity: A Systematic Review. Neonatology 111 (2), 162–171. 10.1159/000448941 27760427 PMC5296887

[B30] Vliegenthart R. J. S. Onland W. van Wassenaer-Leemhuis A. G. De Jaegere A. P. M. Aarnoudse-Moens C. S. H. van Kaam A. H. (2017b). Restricted Ventilation Associated with Reduced Neurodevelopmental Impairment in Preterm Infants. Neonatology 112 (2), 172–179. 10.1159/000471841 28601870 PMC5637296

