# Supplementary material for: Precision Dosing of Doxapram in Preterm Infants Using Continuous Pharmacodynamic Data and Model-Based Pharmacokinetics: An Illustrative Case Series
Source: Front Pharmacol. 2020 May 12;11:665. doi: 10.3389/fphar.2020.00665 (PMC7236770; doi:10.3389/fphar.2020.00665)
Supplement: Supplementary file 1 [file Table_1.docx]

Supplementary Material

# Supplementary Table

**Supplementary Table 1.** Pharmacokinetic parameter estimates of the final doxapram and keto-doxapram models (parent and metabolite).

| **Parameter** | **Final Model: Estimate (SIR RSE %)** |
| --- | --- |
| Fixed effects  CL_DOXAPRAM OTHER ROUTES,i_=CL_DOXAPRAM OTHER ROUTES,p_* (PNA_i_/29)^ƟPNA^ * (GA_i_/25.6)^ƟGA^  CL_DOXAPRAM OTHER ROUTES,p_ [L/h]  Ɵ_PNA_ [exponent for influence of PNA on CL_DOXAPRAM OTHER ROUTES_]  Ɵ_GA_ [exponent for influence of GA on CL_DOXAPRAM OTHER ROUTES_]  V_2 CENTRAL DOXAPRAM_ [L]  Q_1 DOXAPRAM_ [L/h]  V_4 PERIPHERAL DOXAPRAM_ [L]  F_1_ (%)  CL _FORMATION KETO-DOXAPRAM,i_= CL _FORMATION KETO-DOXAPRAM,p_* (PNA_i_/29)^ƟPNA^ * (GA_i_/25.6)^ƟGA^  CL _FORMATION KETO-DOXAPRAM,p_ [L/h]  Ɵ_PNA_ [exponent for influence of PNA on CL _FORMATION KETO-DOXAPRAM_]  Ɵ_GA_ [exponent for influence of GA on CL _FORMATION KETO-DOXAPRAM_]    CL _KETO-DOXAPRAM_  V_3 CENTRAL KETO-DOXAPRAM_ [L]  Q_2 KETO-DOXAPRAM_ [L/h]  V_5 PERIPHERAL KETO-DOXAPRAM_ [L] | 0.645 (13 %)  0.634 (27 %)  5.92 (34 %)  1.54 (48%)  0.103 (23%)  2.18 (22%)  0.735 (13%)  0.115 (22%)  0.377 (23%)  1.96 (50%)  0.256 (21%)  1 (fixed)  0.0443 (60%)  1.24 (38%) |
| Inter-individual variability (eta)  On CL_DOXAPRAM OTHER ROUTES_ [%]  On V_2 CENTRAL DOXAPRAM_ [%]  On CL _FORMATION KETO-DOXAPRAM_ [%] | 50.2 (14 %)  119.6 (20 %)  25.8 (21%) |
| Inter-occasion variability  On CL _DOXAPRAM OTHER ROUTES_ (%) | 55.6 (10 %) |
| Residual variability  Proportional [%]  Additive [mg/L] | 31.3 (3.8%)  0.0035 (19.1%) |

CL_DOXAPRAM OTHER ROUTES,i_=clearance of doxapram through other routes than metabolization to keto-doxapram for an individual patient i, CL _FORMATION KETO-DOXAPRAM,i_=formation clearance of keto-doxapram from doxapram for an individual patient I, V_2_=central volume of distribution doxapram, V_4_=peripheral volume of distribution doxapram, Q_1_= intercompartmental clearance doxapram, V_3_=central volume of distribution keto-doxapram, V_5_=peripheral volume of distribution keto-doxapram, Q_1_= intercompartmental clearance keto-doxapram, F_1_=oral bioavailability, GA=gestational age (median GA=25.6 weeks), PNA= postnatal age (median PNA= 29 days), p=population mean value of a parameter for an individual with PNA of 29 days and birthweight of 1 kg and a gestational age of 25.6 weeks, RSE = relative standard error.
